# ‘An Unimaginable Challenge’: A Cross-Cultural Qualitative Study of Ethics and Decision-Making Around Tracheostomy Ventilation in Patients with Amyotrophic Lateral Sclerosis

**DOI:** 10.1080/23294515.2025.2474928

**Published:** 2025-03-19

**Authors:** Reina Ozeki-Hayashi, Dominic J. C. Wilkinson

**Affiliations:** a Department of Biomedical Ethics and Public Policy, Faculty of Medicine, Osaka University, Osaka, Japan; b Department of Biomedical Ethics, University of Tokyo, Tokyo, Japan; c Institute for the History and Ethics of Medicine, Interdisciplinary Center for Health Sciences, Martin-Luther-University Halle-Wittenberg, Halle (Saale) Germany; d Uehiro Oxford institute, University of Oxford, Oxford, UK; e John Radcliffe Hospital, Oxford, UK; f Murdoch Children’s Research Institute, Melbourne, Australia; g Centre for Biomedical Ethics, National University of Singapore Yong Loo Lin School of Medicine, Singapore, Singapore

**Keywords:** Amyotrophic lateral sclerosis (ALS), tracheostomy, invasive ventilation, cultural comparison, distributive justice, decision-making process

## Abstract

**Background:**

The rate of tracheostomy with invasive ventilation (TIV) for patients with Amyotrophic Lateral Sclerosis (ALS) varies widely. Previous studies have shown that doctors’ values may affect decision-making. There have been no previous international qualitative comparisons of medical decision-making process for TIV or why practice varies.

**Methods:**

We conducted semi-structured in-depth interviews with 16 doctors actively involved in the management of ALS patients from Japan (*n* = 7), the UK (*n* = 5), and the US (*n* = 4). We used three hypothetical cases to explore decision-making. Conversations were transcribed and thematically analyzed.

**Results:**

Our data reveals similarities but also marked differences in views between the US, the UK and Japan. Almost all participants stated that they ought to respect patient autonomy. However, their approaches varied. British participants wanted to (and felt that they should) respect patient autonomy, but they also believed that TIV was not a realistic option. US participants were likely to prioritize patient autonomy over other ethical principles, and Japanese participants were likely to limit patient autonomy indirectly. The option of TIV appeared to be heavily influenced by the availability of healthcare resources in all three countries. The high cost, limited availability and difficulty of treatment meant that particularly in the UK and the US, it is challenging to receive TIV even if patients wanted this.

**Conclusions:**

Our study illustrates how the emphasis on autonomy varies along with variations in the way care is organized in the setting of highly resource intensive treatment and progressive severe disabling illness. There is a need to review elements of the decision-making process in all three countries. This includes the need for transparent, ideally centralized, decision-making guidelines about the provision of TIV. Although we investigated a rare neuromuscular disease, our results will be relevant to other diseases requiring highly resource-intensive treatment toward the end of life.

## Introduction

Amyotrophic lateral sclerosis (ALS) is a neurodegenerative disease that, as the disease progresses, makes it difficult for patients to breathe on their own. When patients with progressive ALS develop respiratory failure despite noninvasive support, they will likely die if they do not receive Tracheostomy with Invasive Ventilation (TIV). However, the proportion of ALS patients receiving TIV varies significantly from country to country. In the USA, where the rate of TIV is 1-15% (Connolly, Galvin, and Hardiman 2015), guidelines state that TIV can be considered as an option to maintain quality of life in ALS patients who wish to be on long-term ventilator support (Miller et al. 2009), but most medical insurance companies do not cover it. In the UK, TIV is rare (Connolly, Galvin, and Hardiman 2015), and treatment guidelines do not mention it as an option. Japan has the highest rate of patients with TIV in the world (27-45%) (Connolly, Galvin, and Hardiman 2015). Potential reasons for this include the fact that TIV is covered by public medical insurance, patient organizations recommend TIV (Rabkin et al. 2013), and once treatment is started, it is difficult to discontinue.

TIV is not a curative treatment for ALS, and even with TIV, there is a risk that the disease will progress and eventually lead to a Locked-in Syndrome where patients are paralyzed and unable to communicate. However, although small, some data shows that quality of life can be acceptable (Kaub-Wittemer et al. 2003; Marchese, Lo Coco, and Lo Coco 2008; Vianello et al. 2011), and it is possible that TIV may provide some benefit for some patients. The decision whether or not to embark on TIV is potentially difficult for both patients and doctors. However, there have been few studies investigating how physicians explain the issue to patients, their attitude toward the discussion and the difficulties they experience during such discussions. A previous short survey of physicians in Japan and the US showed that physicians felt conflicted: they would not want to receive TIV themselves if they had ALS, but most Japanese participants recommended TIV to their patients (Rabkin et al. 2013). There are potential cultural differences. In the US and Japan, treatment without patient consent is considered unlawful under civil law. However, it is unclear whether the Japanese court would sanction the withdrawal of life-sustaining treatments even where patients have consented to this (Ozeki-Hayashi et al. 2023). There have been two attempted criminal prosecutions relating to TIV withdrawal. In the first case, where treatment was withdrawn without explicit patient consent, the high court sentenced the physician to one year in prison with three years of suspension. The latter case (in which consent in seven patients was unclear) was ultimately dropped due to insufficient evidence (Tanaka et al. 2020). But both cases potentially make Japanese doctors worry about facing legal or social sanctions over forgoing TIV (Ogino 2010). There is a Japanese guideline (not tested in the courts) that permits discontinuation of treatment in patients at the end of life with adequate Shared Decision Making (Ministry of Health, Labour and Welfare 2007). But it is still controversial in Japan whether ALS patients who require TIV should be diagnosed as having reached the end of life. It means that patients may practically be unable to stop TIV even if they wish, due to doctors’ fear of prosecution (The Japanese Society of Neurology 2023).

On the other hand, some papers indicated that the patient’s decision-making process is influenced by the doctor’s or family’s wishes (Meininger 1993; Moss et al. 1993). Some ALS patients in Japan have reported deciding to receive TIV based on their family’s wishes (Mori and Yuasa 2006). In another Japanese article, doctors’ positive and negative attitudes toward TIV sometimes conflicted with the patient’s decision about TIV (Kitamura et al. 2002).

We aimed to explore the attitudes and values of Japanese, US and UK physicians on TIV decision-making process for ALS patients. Comparing the decision-making processes of ALS patients in different cultural and economic contexts, may help clarify the ethical challenges in decision-making.

## Method

### Participants

We recruited twelve doctors in the three countries who had treated ALS patients and participated in decision-making about TIV. Snowball and purposive sampling were used to recruit participants from tertiary institutions with a range of experience and backgrounds. Participants included neurologists (in Japan, the UK and the US), palliative care physicians (UK and US) and a respiratory doctor (UK) (Table 1). Participants received a description of the research in advance, and provided informed consent to participate. Participants could withdraw at any time. The study and all protocols were approved by the University of Tokyo Graduate School of Medicine Research Ethics Review Committee (Review number: 2022243NI) and the University of Oxford Research Ethics Review Committee (Review number: R84248/RE001).

### Data collection

Data were collected between December 2022 and August 2023 using semi-structured interviews; All participants were given three hypothetical cases (Table 2) and asked how they would approach the case if they were the attending physician. For each case, they were asked the same questions in the interview guide, developed based on a literature review on the decision-making process for TIV in ALS (Ozeki-Hayashi et al. 2022) (Table 3). Interviews were conducted by a single researcher (ROH), audio recorded and subsequently transcribed verbatim for analysis. The interviews for Japanese participants were conducted in Japanese and those for others were conducted in English. Japanese interviews were translated into English by ROH. Considering that the language used in the interview could affect the responses, the interviewer explained the meaning of the questions carefully and tried to clarify the participants’ responses when the interviews were given in English. Furthermore, as the coauthor (DJW) is a native English speaker, both researchers repeatedly examined the interpretation of the participants’ words based on the transcripts.

### Data analysis

Qualitative analysis was conducted using thematic analysis to categorize individual doctors’ attitudes and behaviors and generate themes. This is a systematic process used to find patterns in qualitative data (Boyatzis 1998; Braun and Clarke 2021). One of the authors read the transcripts, conceptualized the information by relating it to participant concerns and attitudes toward decision-making, and coded the data to prevent the loss of meaning or distortion of the content of the sentences, phrases, and words. Codes highly similar in content among the multiple codes gathered were merged to generate subcategories. Codes were further grouped according to their content to make categories with higher levels of abstraction. The first author discussed the coding process regularly with the second author. They discussed the interpretation of the participants’ narrative, including their way of thinking, clinical judgment, and each country’s culture and medical system. As the first author (who has a Japanese cultural background) was aware of the potential for bias or misunderstandings in interpretations, the discussion with the second author (who has a Western cultural background) clarified and verified interpretation to ensure accurate coding. To further ensure the validity of the analysis, we had multiple peer debriefing sessions (Morse 2015). Peer debriefing is a process in which researchers who are not directly involved in the research, but who are familiar with medicine, philosophy, and ethics and education, discuss the analyst’s interpretation of interview data to find any important issues that the analyst had not noticed. In this study, which is a cross-cultural comparative study, we repeatedly discussed whether there were any problems with the analysts’ interpretation of the cultural context of the interview data. The study used Reflective Thematic Analysis (Braun and Clarke 2021), with data collection terminated based on the principal researcher making an interpretive judgment of whether a sufficient depth of understanding had been reached to understand the phenomenon and answer the research question. Theoretical saturation was not sought. Qualitative analysis software (NVivo 14) was used in the series of coding processes.

## Results

Two broad themes and related categories were identified in the interviews. These themes related to medical considerations and decisions of TIV, and also the participants’ culturally-constrained norms. All themes and categories are presented in Table 4 and 5.

### (1) Balancing the benefits and burdens of TIV for ALS patients

#### 1-a Therapeutic applicability of TIV for ALS patients

Participants gave different assessments of the disease stage and prognosis of the hypothetical patient requiring TIV in the case examples. Some participants said that they would not be surprised if the patients died at any time (i.e., that they were near the end of life) and that even if such patients started TIV, their life expectancy would be six months to a year. On the other hand, one doctor said that such patients are not at their end-of-life and they could live ten years or more if they received TIV.

“I think that [the outlook for] these people [is] very, very clear. They have ALS… are not going to live for 7 to 10 years. They’re going to live for six months.” (UK1)

“A patient with TIV can live more than ten years; that is, he is not at the end stage of his life. The most common definition of end-stage means a half-year prognosis or no expectation for improvement, therefore, he is not at the end stage.” (JP7)

However, even if they thought that TIV could help the patients live longer, many participants said that they did not consider TIV as a standard treatment for ALS patients.

TIV is not a standard treatment option, it’s not like wearing glasses for bad eyesight. (JP6)

As a reason for this, many said that the QOL for patients with TIV appears very challenging, and the burden on the family is considerable.

“Life with TIV looks like a nightmare, unfortunately in some cases. It’s not easy, and both the patients and their families will suffer terribly. For example, we had a case… a caregiver (the patient’s mother) turned off the TIV of her son with ALS and she also tried to kill herself, but she failed and was found guilty of murder.” (JP4)

“I think that, if there were a huge burden to, like, if the parents did not help at home, it would be a much bigger lift for the parents, physically, emotionally, potentially, financially, I mean, they would definitely, if they didn’t have the public health insurance covering this even just having a ventilator in the home, and all the supplies can become very expensive, and financially drain a family.” (US4)

Some also questioned the indication of TIV for ALS patients, which has no curative treatments.

“If ALS were curable, I think TIV would be a fine option. But if there is no hope for cure, then I don’t think we should apply TIV for ALS patients proactively.” (JP1)

#### 1-b TIV as a scarce medical resource

Furthermore, interviewees spoke about TIV as a medical resource. Japanese participants mentioned TIV as a feasible and available option for ALS patients because of financial support from medical insurance and policy relating to intractable disease (Ministry of Health, Labour and Welfare 2014) which means that are no allocation restrictions on the availability of TIV. In contrast, British and American participants explained that even if patients wanted TIV, it was difficult to access, not only financially but also because of limited availability of this treatment.

“In Japan, of course, it costs money, but the patient can use TIV without much money.” (JP6)

“So, we very rarely even get into serious conversations about tracheostomy ventilation. It’s largely not available. If someone asks, it isn’t likely to end up happening.” (UK4)

Moreover, several participants pointed out that society needed to deal with this problem of how to allocate health care resources.

“I think that’s a huge problem just in society in general and not something that one person can fix in one medical interaction. These are very personal decisions (for TIV), but they’re also very much honoured by the social narrative that we all live within, where there’s like an expectation that everyone is a very independent, productive member of society.” (US1)

### (2) Doctor’s attitudes and culturally-constrained norms for TIV decision-making

#### 2-a The relationship between the patient and the others in the decision-making process

American and British interviewees clearly gave the view that patients have a right to refuse treatment. Therefore, they said would be unlawful for them not to respect the patient’s refusal.

“A patient has a right to refuse any medical intervention. And if a patient does refuse medical intervention, as long as it is their capacitous decision, then healthcare professionals have to obey that and to not do so would be illegal. It would be an assault to continue to give an intervention that the patient had decided he did not want.” (UK5)

On the other hand, Japanese participants stated that the final decision to decline treatment is not the patient’s alone to make. Some talked about the patient’s right to refuse the treatment not being legally supported in Japan.

“We will make a decision based on not only the patient’s wish but also on others’ wishes who are related to him…Under current Japanese law, if we withdraw TIV, it is still regarded as assisted suicide. In other countries, it might be different, but in Japan, stopping TIV is still illegal in my understanding.” (JP1)

For the British and American interviewees, the family had an essential role in providing information about the patient, but the family members’ own wishes for the patient were not directly relevant.

“[We ask them]…’What can you tell me about the patient that will help me to make the right decision?’… not ‘what do you want me to do for him?’.” (UK4)

#### 2-b Patients’ decision-making capacity and competency

There were differences between British, US and Japanese participants in their views about patients’ decision-making capacity. Japanese participants stated that the decision-making capacity of ALS patients was unreliable. In contrast, British and American participants mentioned using a formal method to assess decision-making capacity.

“Some ALS patients tend to change their personality or lose their cognitive function. I am worried whether their decision of withdrawing TIV at that time reflects the patient’s authentic desire.” (JP4)

“You can assess the patient’s ability to understand the information you’re giving them to make an informed decision, and check that they remember the decision and that it’s consistent. So, people can have cognitive impairment, but depending on the level of cognitive impairment, it won’t necessarily affect their ability to make a capacitous decision. So, you do a capacity assessment that is decision-specific. There is a capacity assessment framework that we would use to ensure that the individual, regardless of their cognitive abilities, …[has] capacity to make the decision.” (UK5)

#### 2-c Doctor’s communication and attitude at the decision-making process for TIV

Some participants said that their communication style was “Shared-Decision-Making” (SDM), but they thought each doctor provided SDM differently.

“I think that shared decision-making is the gold standard here. But I will acknowledge that different physicians may practice differently.” (US3)

Most participants said they would discuss treatment options and disease progress for ALS patients from an early phase.

“And I talk a lot about with the patient and the family about having an open communication about that, and I think it’s really important to have a conversation before the intervention is even done.” (US4)

However, the communication style differed between participants. Participants from the US suggested that US doctors often prioritize patient autonomy, communicate all treatment plans equally and try to take a neutral stance on each option.

“…It’s more like the patient or the family are … the customers, and they get offered all these options. …I just think in the US, often, a lot of the burden is put on the family in terms of, … navigating all these options and no one’s going to tell you what the right one is.” (US1)

Most British participants said they would not give patients the option of TIV during discussions unless the patient mentioned it. Even if they were to discuss this option, they believed that TIV was not feasible since it was not available within the National Health Service; consequently, talking about it to the patient would be unhelpful.

“That automatically starts the conversation because you’re saying … respiratory muscle weakness…is why people with ALS die, but we’re going to help your symptom[s] with the non-invasive equipment.” And then most people at that stage, if they say, ‘What happens if I get worse?’ and we said ‘Well, it will get worse, and ultimately, even the NIV will not keep you alive.’ … you could argue that maybe we’re avoiding [tracheostomy] discussion sometimes. (UK1)

“Most British people do not wish [for] TIV…But even if [a] young patient is very keen on TIV…., it’s not going to happen. It’s not that we’re a very poor country. Our health service does have some big problems, but it’s because it’s a national scheme, it (TIV for ALS) doesn’t involve insurance.

There’s no pathway to plan ahead…I don’t talk about that (TIV option) first, because I think when people are thinking about tracheostomy, it’s unhelpful to start off with… (explaining that patients) will never get it, it’ll never happen. That’s really unhelpful, because sometimes then it makes them want it (TIV) even more.” (UK3)

Many Japanese participants said that it was important to fully explain the burdens of TIV, even though this is an available option in Japan. In addition, they said that it is critical to clearly ask the patients’ purpose for wanting TIV. Due to the lack of legal support, withdrawing TIV is not feasible in Japan. Some participants would not allow TIV unless the patients understood that later stopping ventilation would not be possible.

“I always told the patients that it might be an unimaginable challenge they have to live with TIV, depending on others for a lot of things in their lives. So, I asked them to decide carefully. It’s challenging. For instance, the patients need family support, and they have to be hospitalised if the family can’t care for them. In such cases, they might have to move to a different hospital every six months due to the medical insurance. …Even though the patients are all covered by health insurance, there are some rural areas that have fewer support service than urban areas. The services provided with public financial supports depend on the policies of the local council.” (JP4)

“Before starting TIV, I always ask the patient to confirm that they [understand that] will never be able to stop TIV, even if they really want to.” (JP2)

Other Japanese participants noted that in many cases, patients are greatly influenced in their decisions by doctors’ or hospitals’ beliefs about TIV.

“It largely depends on the hospital or the doctor for the patients to get TIV. Sometimes, in a certain hospital, almost all ALS patients are receiving TIV; in contrast, in another hospital at present, almost no ALS patients receive TIV. It might relate to a doctor’s positive or negative attitude or explanation about TIV, and in some cases, it might be influenced by the hospital policy. It means that the hospital or doctor’s policy mainly decides the TIV for ALS patients in Japan. I think it’s problematic.” (JP7)

#### 2-d Doctor’s concerns regarding cultural constraints on TIV withdrawal

Most US and UK participants recognized that withdrawal of TIV was ethically equivalent to withholding TIV. However, some UK and US participants talked about concern that patients may misunderstand withdrawing TIV as euthanasia or assisted dying. One UK interviewee stated the necessity for clarifying patients’ understanding of such differences because euthanasia and assisted dying are illegal in the UK.

“But with that caveat, it’s not guaranteed when you remove it that somebody will necessarily die quickly, and they need to understand that, really. And I think certainly we wouldn’t want to do it if they were stating that the reason was that then they would be able to decide when they died. We’d have to sort of talk about that and understand what it was that they were worried about happening.” (UK3)

Even where Japanese participants expressed a desire that the patients have the legal right to refuse treatments, they stated that they could not consider this issue in the same way as other countries because currently withdrawing TIV was perceived by society as murder or assisted suicide. Some participants were worried that expressing a position of agreement with stopping TIV would lead to the misunderstanding by society that those doctors were “denying the value of the lives of other patients living with TIV.”

“If I agree with the patient’s wish to withdraw TIV, it might socially mean I give messages to the other patients trying to live with the same suffering, as if I think their lives are not worth living.” (JP4)

## Discussion

This study is the first international qualitative comparison of physicians’ attitudes on TIV in ALS patients. While the study builds on previous quantitative surveys (Rabkin et al. 2013), our study was able to investigate doctors’ experiences in depth. Our data reveals some similarities but also marked differences in views on prognosis, treatment appropriateness and decision-making in ALS between the US, the UK and Japan.

### Shared view on mechanical ventilation: technically feasible, but undesirable

Most participants said that decision-making about TIV is challenging and demanding. They considered ALS as a progressive neurodegenerative disease, and therefore, did not view TIV as a standard treatment option.

Furthermore, interviewed doctors referred to the considerable burden of TIV in terms of patients, families, financial and medical resources and they argued that family and caregivers should be considered when making treatment decision. This is consistent with recommendations from Norwegian neurologists and colleagues, who argued that the burden on spouse and children should be a compulsory part of the information given before the choice of TIV is taken (Magelssen et al. 2018).

Consistent with Rabkin’s survey, in all three countries, the interviewees said that they did not think TIV was a desirable treatment, and volunteered that they would not want to receive TIV if they personally had ALS (Rabkin et al. 2013).

### Respecting and disrespecting patient autonomy: divergence and conflict

Almost all participants stated that they ought to respect patient autonomy and some explicitly referred to their approach as “shared decision-making (SDM)”. However, the nature of their approaches and ethical tensions at the decision-making process were varied. In previous studies, it was reported that physician’s understanding of their normative obligations in SDM varied between doctors (Landry 2021); our result seemed to be also consistent with this research.

British participants wanted to (and felt that they should) respect patient autonomy, but they also believed that TIV was not a realistic option because it was not readily available. Moreover, they did not believe that TIV was beneficial for the patient. In other words, they wanted to respect patient autonomy but giving the option of TIV posed a potential threat to beneficence. As a result, they took the approach of not giving TIV option unless the patient mentioned it. In a review article in 1998, Borasio et al. noted that UK physicians were reluctant to recommend or consider TIV for ALS patients, but were increasingly mentioning TIV as a genuine option due to the shift for patient-centered approach (Borasio, Gelinas, and Yanagisawa 1998). However, our results suggested that British participants appear to be returning to their former, more paternalistic approach. In the UK, the judicial decision (McCulloch v Forth Valley Health Board 2023) held that there is no legal duty for a doctor to present treatment opinions that they do not regard as reasonable, as long as the doctor’s view (about what would be appropriate) is supported by a reasonable body of medical opinion (Sokol 2024). British participants in our study might hold views reflecting this legal development. Although UK interviewees defended not offering TIV option, claiming that “British people do not want TIV (UK1)”, there is a lack of data on the views of patients with TIV and families regarding TIV or QOL with TIV in the UK (Wilson et al. 2023).

American participants mentioned autonomy as the most significant ethical principle in the US. They, therefore, felt as though they had an obligation to explain all options to patients, including TIV. They appeared to believe that, to respect patient’s self-determination, they should provide sufficient medical information, not necessarily their recommendations of treatment options. In previous study, although more than 70% of the US participated doctors stated that they would provide treatment options along with their own recommendations in making treatment decisions for ALS patients, participants in our study appeared more reluctant to offer recommendations (Rabkin et al. 2013).

Finally, many Japanese participants wanted to (and felt that they should) respect patient wishes and explain all options, including TIV, in a value neutral perspective. However, their perceived societal expectation for doctors to prolong life, the social imperative arising from ALS patient advocacy groups explicitly endorsing TIV, and concerns about the lack of legal support to withdraw TIV once they start, may have influenced their attitude significantly in the decision-making process. For instance, if doctors thought TIV would not be feasible to the patient based on factors such as the lack of patient’s family support or where they thought that the patient would probably ask for withdrawal of TIV in the future, they highlighted the drawbacks and burdens of TIV at the decision-making process. It means that their approach became more paternalistic. These responses were similar to, but more complicated than Japanese participants in Rabkin’s study, most of whom recommended TIV to their patients (Rabkin et al. 2013).

### The challenge of resource allocation: shared, but diverging views

The option of TIV appears to be influenced by the availability of healthcare resources in all three countries. The high cost of treatment and difficulty accessing it meant that particularly in the UK and the US, it is challenging to receive TIV even if patients wanted this.

In the UK, treatments for patients with ALS (as for other illnesses) are assessed for their effectiveness, and (if found to be beneficial at a reasonable cost) made available through the publicly funded health care system. Guidelines by the National Institute for Health and Care Excellence recommend early discussion with patients with moter neurone disease (including ALS) about the option of noninvasive ventilation, and that this should offered where there is respiratory impairment (Motor Neurone Disease: Assessment and Management 2019). Strikingly, however, there is no discussion in those guidelines about invasive ventilation. It appears, as a consequence that at the present time TIV is not a realistic option for patients with ALS.

In the US, patients’ access to healthcare resources depends on whether or not they have a particular medical insurance. It was noted by interviewees that Medicare, Medicaid and most insurance companies would not fund this treatment (this was also supported in English et al. (2023)) and on top of that, the availability of TIV option is severely limited by the potential resources of the individual (i.e., that this may be an option only for those with considerable financial means).

On the other hand, TIV is an available option in Japan covered by medical insurance and most Japanese participants did not mention medical resource allocation. Neither is this mentioned in treatment guidelines in Japan (The Japanese Society of Neurology 2023). This may be influenced by the fact that public discussion of medical resource allocation in Japan has led to protest letters submitted by advocacy groups for patients with disabilities (Japan National Assembly of Disabled Peoples’ International (DPI-Japan) et al. n.d.)

Still, despite the greater accessibility of TIV in Japan, Japanese interviewees acknowledged that availability of TIV for ALS patients depends on whether patient families can support it. Without family support, patients might have to be hospitalized for a long time and may be transferred from one hospital to another. They mentioned disparities in healthcare resources between urban and suburban areas also limit access.

### Ethical implications and suggestions for the decision-making process for TIV

Our interviews highlight the considerable challenges in decision-making, but they also point to some potential lessons.

### Respecting autonomy

Our interviewees were aware that decisions about invasive ventilation were very difficult for patients. That is because such treatment is technically possible and can prolong life, but is highly burdensome. The physicians worried that patients may choose this option to the detriment of their care in the last phase of life.

UK participants tended toward more paternalistic attitudes, with doctors withholding information about the option of TIV partly on the basis of the patients’ best interests. But at the other end of the spectrum, the US participants described a medical culture with perhaps too much attention to autonomy. They referred to a tendency to offer options, without offering advice.

In one way, the Japanese approach appears ethically preferable, in that there is open discussion with patients about all options, even if those options are not necessarily something the physician would recommend. It was striking and instructive that some of the Japanese interviewees described challenging and testing patients’ choice of TIV – to ensure that they understood the consequence of their decision.

However, the UK/US approach was much more strongly supportive of patient choice to *not* embark on TIV and in particular to respect patients’ decision to *discontinue* TIV. Here, the approach described by the Japanese physicians appeared to be much more problematic. For commencing TIV, the physicians reported reluctance to follow patient’s preferences due to concerns about patients’ capacity (even without explicit assessment of this). There was significant deference to family wishes. It was also clear that patients’ autonomy in Japan was highly limited when it came to stopping TIV.

Addressing this latter problem may require clear professional guidance or potentially legal change in Japan. One possibility would be to draw on international consensus about the nature of this illness. Interviewed doctors in the UK and the US all regarded patient with ALS and respiratory failure to be “dying” or “in an advanced terminal state.” If Japanese doctors were to refer to diagnostic criteria such as those used in the UK and US, they might then be able to apply guidelines that permit withdrawal of end-of-life ALS patients’ TIV following discussion with patients (Ministry of Health, Labour and Welfare 2007). It may be, for example, that professional guidelines in Japan could recommend referring all patients with ALS who develop respiratory failure to palliative care teams. The potential need for TIV could be taken as a marker of the end stage of this progressive illness with support for withholding (or withdrawing) treatment.

Alternatively, treatment could be provided potentially as a therapeutic trial. Where patients are unsure about whether their quality of life would be acceptable, or whether they could manage the burdens of treatment, this could be offered for a strict time-limited period with a plan to review, and discontinue if not judged beneficial at the end of that period. It is remains to be seen whether such treatment trials would be acceptable to Japanese patients and families.

### Highly resource-intensive treatment

Participants in the three countries were conscious that TIV is a highly resource-intensive treatment. Such treatment necessarily raises important issues of healthcare resource allocation and might not be available even if it would be beneficial or desired by patients. A review by Strech et al. (Strech, Synofzik, and Marckmann 2008) found psychological distress and frustration among doctors in undertaking rationing of scarce resources at the bedside. This means that there is a potential psychological burden for doctors engaging in discussions between patients and doctors about TIV.

However, as highlighted by participant US1’s narrative, TIV decisions are not only very personal but also raise the problem of how society respects each individual. This is not a problem that can be solved in private interaction between health professionals and patients. There is a need for transparent, ideally centralized, decision-making about the provision of highly resource intensive therapies. Such guidelines would need to incorporate explicit, transparent criteria including ethical considerations and be fully discussed with stakeholders.

In the UK, there is a process for such decisions, and indeed there is guidance in relation to noninvasive respiratory support. However, there is no guidance in relation to TIV. If the UK determination is that resources are insufficient to offer this option, that should be made clear so that patients are aware of the basis for decisions (and could seek treatment if desired and they have the resources to do so). If the judgment is that TIV is not beneficial, that should be articulated (and defended).

In Japan, the treatment is made available, but there is no clear policy in relation to this (nor, as noted above, in relation to stopping treatment). There was some discussion of variability in access to TIV in different areas of the country.

Variability in access was a significant challenge in the US, with a comment that few patients would be able to obtain treatment. There is a need to consider whether this is a treatment option that should be publicly funded (as is the case in the US for another expensive life-prolonging treatment: renal dialysis (Mendu and Weiner 2020)). If it cannot be made available, patients with ALS and respiratory failure should be supported to access other treatment options (especially palliative care).

Developing guidelines for TIV will be challenging. However, these guidelines should as a minimum include recommendations that doctors inform patients of all options, including that of TIV. Even if an individual patient is not able to access it, we believe that patients have the right to know that TIV is a theoretical option in their circumstances. On top of that, doctors should be prepared to share their professional recommendations (given the patient’s social situation and medical condition) to help ease the decisional burden. This could include a time-limited trial of TIV where patients are uncertain if TIV would be beneficial.

### Limitations and strengths

Our study was able to captured perspectives among specialist doctors in three countries with different cultural and legal backgrounds and very different rates of initiating this therapy. It was able to identify the distinct ethical conflicts doctors face in TIV decision-making in different settings.

There are several limitations. We do not include the views of other healthcare professionals, patients, and patient families, all of whom are involved in actual TIV decision-making. Future cross-cultural research into these important stakeholders’ views will help us better understand the decision-making process.

Our small sample size limited our ability to explore all features of the doctor attitudes regarding the ALS decision-making process. Further insights or questions may have emerged with additional interviews or with deeper probing. We deliberately chose three countries with contrasting patient care practices. Perspectives of physicians in other countries may, or may not, overlap with those we report here. A larger international study would clarify the dilemmas experienced by doctors involved in ALS decision-making.

In addition, given that the topic of our study dealt with what could be described as socially sensitive material - it is possible that doctors may be giving perceived socially desirable answers that differ from their own true beliefs. Also, the participants answered their opinions on hypothetical case scenarios. In a real clinical practice, their decision-making could have involved a more nuanced process of discussing the patient’s wishes and needs, exploring potential psychological and physical treatments and improving symptom management.

## Conclusions

Our study provides insights into physicians’ ethical conflicts and the contrasting and overlapping ethical challenges of decision-making for patients with ALS requiring TIV. The medical care in the three countries included in our study includes a spectrum from TIV being rare to it being widely available. Our interviews identified the underlying ethical assumptions and norms in the UK, US and Japan that helped to explain differences in practice. They highlight the challenge of communication, and of reconciling and respecting patient autonomy and distributive justice when it comes to providing highly resource intensive treatment in the context of terminal and progressive illness. Although we have focused on a single, relatively rare neurological disease, the findings potentially may be relevant to a wider group of patients with neurodegenerative conditions and respiratory failure. We have suggested some important ethical lessons for the care of patients with ALS in these countries, but potentially also with other illnesses and in other parts of the world.

## Figures and Tables

**Table 1:** Participants characteristics.

ID	Gender	Age	Specialty
JP1	Male	30s	Neurology
JP2	Female	40s	Neurology
JP3	Male	50s	Neurology
JP4	Male	60s	Neurology
JP5	Male	40s	Neurology
JP6	Male	50s	Neurology
JP7	Male	50s	Neurology
UK1	Male	50s	Neurology
UK2	Female	50s	Respiratory
UK3	Male	50s	Neurology
UK4	Male	50s	Palliative medicine
UK5	Male	50s	Neurology
US1	Female	30s	Neurology
US2	Male	40s	Neurology & Palliative medicine
US3	Female	30s	Neurology & Palliative medicine
US4	Female	40s	Neurology

**Table 2:** Hypothetical cases.

A 29-year-old man was diagnosed with Amyotrophic Lateral Sclerosis (ALS) six years ago. For the first 4 years following diagnosis, the progression of the disease was slow, and he was able to move his body with some difficulty. Two years ago, his condition accelerated.			
**[Case1]**	**[Case2]**	**[Case3]**	
He became bedridden and had trouble breathing on his own. He decided to receive Noninvasive ventilation support (NIV). **He, his family, and his care team** **held several discussions on his future treatment** **and disease trajectory, and he decided to receive** **a tracheotomy and invasive ventilation (TIV) and** **percutaneous gastric feeding tube (PEG).**	**One day he suddenly lost consciousness in the** **hospital during his regular visit. When he** **regained consciousness, he found he had** **been put on a TIV and a percutaneous gastric** **feeding tube without his consent. He asked to** **withdraw from TIV, but his doctor refused his** **request citing legal concerns, and his family** **told him he should continue with TIV.**	He, his family, and his care team held several discussions on his future treatment and disease trajectory, and **he** **refused to receive a** **tracheotomy and** **ventilation (TIV). His** **parents did not agree** **with him. **His parents have not been burdened with his care to any great extent. All medical care and nursing services are covered by public health insurance, and he has no financial problems.	
He has been hospitalized for a short periods two or three times per year due to pneumonia or urinary infections. He can stay at home almost all the time. He can move his eyes, so he uses a computer with eye input to communicate. His parents live with him, and he has 24-h nursing care from a home care professional team. His parents have not been burdened with his care to any great extent. All medical care and nursing services are covered by public health insurance, and he has no financial problems. One day, he asked his attending doctor to stop all his treatments. He said, “I want to end this fight. Enough is enough. Please let me die.”			
	His parents have not been burdened with his care to any great extent. All medical care and nursing services are covered by public health insurance, and he has no financial problems.		
	However, he asked his attending doctor to stop all his treatments again. He said, “I want to end this fight. Enough is enough. Please let me die.”		

**Table 3:** Interview guide.

What do you think about the ethics of this decision? And the law? People might use different words or phrases to describe what is going on when the ventilator is withdrawn in a case like this. How would you describe it? Tell me about your response to this case? How would you feel if you were the doctor? Some people have said that “physicians are ethically obligated to do everything reasonable to keep patients alive unless the patient is terminally ill and near death”. What do you think about this? Some people think “that patients have a right to refuse treatments they do not want to receive, even if the doctor disagrees?” what do you think about this? In your view, when is it ethical to stop life-sustaining treatments?(Is there a different meaning for withdrawing ventilation from other neurological diseases?)

**Table 4:** Theme1 Views on balancing the benefits and burdens of TIV for ALS patients.

Category	Subcategory	Quote
1-a Therapeutic applicability of TIV for ALS patients	TIV for ALS as a standard treatment	TIV does not stop disease progression, so I would not explain it as a standard treatment. (JP6)
		I’ve got much less experience of people with tracheostomy ventilation in MND and you know what strikes me is that of course the ventilation doesn’t stop the other things happening which can end the person’s life. (UK4)
	Survival expectation for ALS patients requiring TIV	I think that these people very, very clear, they have ALS, are not going to live for 7 to 10 years. They’re going to live for six months.(UKI)
		I say that with a ventilator we can extend that to a decade or more depending on how things are going with your health and other comorbid conditions. (US3)
	Disease stage assessments for ALS patients requiring TIV	I think ALS patients requiring TIV are not at the end stage of life. (JP2) I would describe it as this young man is now dying of ventilatory failure caused by amyotrophic lateral sclerosis and up until now the ventilation has succeeded in delaying that but that’s what’s happening. (UK4)
	QOL of ALS patients with TIV	TIV will help the respiratory function for the patients, but they will be suffering from other symptom. (JP1)
	Family burden for ALS patients with TIV	I am worried about the family, because they will be exhausted to care the patient with TIV. Sometimes, I had the patient family relationship broken. (JP5) And so when families provide that care themselves, it’s very high intensity hybrid and their whole lives become about providing this ventilator care and when they hire someone else to do it, it becomes really financially burdensome. (US3)
1-b TIV as a part of scarce medical resources	Availability of TIV for ALS Restriction of TIV use due to lack of medical resources	Japanese patients have feasible options for TIV (JP4) One of the major ethical issues we have is when sometimes people want to have things like tracheostomy and ventilation, but we don’t have the resources. (UK3) I would say that there are some patients who can go home in a patient like this. But they are the patients that are more affluent who have the means to pay for home nursing at home. (US2)
	The responsibility for the society to decide how to allocate medical resources	This is a very complex and troublesome project. It’s not simply, “Let’s find some treatments and cure ALS,” which is the way patients think about it and the way that funding bodies think about it and the way society thinks about it. The reality is we are entering on a journey where we will make more people disabled for longer. That may sound negative, but that’s the reality. This is, it’s not my opinion. It is a fact. We have to deal with that fact. (UK1)

**Table 5:** Theme2 Doctor’s attitudes and culturally-constrained norms for TIV decision-making

Category	Subcategory	Quote
2-a The relationship between the patient and the others in the decision-making process	Patients’ right for decision-making	So, a patient has a right to refuse any medical intervention. And if a patient does refuse a medical intervention, as long as it is their capacitous decision, then we as medical professionals, healthcare professionals have to obey that and to not do so would be illegal. It would be assault to continue to give an intervention that the patient had decided he did not want. (UK5) I think if it’s his capacitous decision and we’re clear that he’s making it in a capacity way, I think we can just do without the consent of the family or assent of the family. (US5)
	The role for the patient family in decision-making	And occasionally we see mistakes made where people just follow blindly what the family say but I think we also often see mistakes made where people disregard what the family say and fail to notice that they’re actually telling us something really important about the person in the bed... What can you tell me about the patient that will help me to make the right decision, not what do you want me to do for him. (UK4)
2-b Patient’s decision-decision making capacity and competency	The doctor’s concern for the decision-making capacity of ALS patients requiring TIV	Even if some research said most ALS patients did not lose cognitive function, I had some patients with mild cognitive dysfunction. Moreover, the longer they suffered ALS, the more they got the mood disorder or mild depression. I can’t believe they are competent for the very difficult decision making for withholding TIV at any point of their disease. (JP1)
		It is natural for humans to change or fluctuate their wishes. I can’t believe ALS patients can decide firmly. Because it might change later. (JP5)
	The patient’s decision-making capacity assessment system	Yeah, so that’s important. That’s why you need to make sure that he has capacity. So, capacity in the UK you don’t need to do a cognitive assessment to assess capacity you assess their ability to understand the information that you’re giving them to make an informed decision, check that they’re remembering the decision, that it’s a consistent decision. So, people can have cognitive impairment, but depending on the level of cognitive impairment, it won’t necessarily affect their ability to make a capacitous decision. So, you do a capacity assessment that is decision specific. Yes, each decision has its own, you would check capacity, particularly for such an important decision, you would check they have the capacity and there is a capacity assessment framework that we would use to ensure that the individual, regardless of their cognitive abilities, that they have capacity to make the decision. (UK5)
2-c Doctor’s communication and attitude at the decision-making process for TIV	The process of decision-making for starting and withdrawing TIV	I wouldn’t just say " Okay, Let’s do it." I would say this could have major consequences, meaning that you could die pretty quickly. Some people find over time they’re much more able to accept this new quality of life. What I would recommend is we try this for a little longer and revisit. (US4) Yes, so I would use the same framework that I’ve described. It wouldn’t be a quick, like I wouldn’t do it then and then. There would be a whole process we go through but if at the end of that process that we were certain that he was communicating clearly a capacitous decision and was not under any undue influence, then yes, we would manage him the same way. (UK5)
	Holding the TIV option back to the patient	I mean, we didn’t say, "Well, therefore, would you like a tracheostomy?" Because we respond to what the patient is saying. I mean, you could argue that maybe we’re avoiding that discussion sometimes. But it comes from I think a deep understanding and knowledge of the disease. And having looked after many, you know, several thousand patients, I feel I can instinctively go with the patient on that sort of journey and understand what they want. I mean, if they want a tracheostomy, we have that discussion, but we don’t have any kind of systematic way of triggering the discussion if that’s what you’re asking. (UK1)
		I don’t tend to say, do you want NIV 24/7 or not, that’s too threatening. (UK2)
	Providing the TIV option to the patient with the detailed information about the hardship of the life with TIV	In the US, a few famous ALS patients with TIV are always active, young and positive...most of them are wealthy... however, not all the patients with TIV can be like that, the patients should know that. (JP6)
	Requiring the patient’s firm purpose to start TIV	I don’t want hold on to my life with TIV. ("Do you think that the patient with TIV hold on to their life?") Yes, they tend to have a firm belief for their life with TIV. However, if the reason is to see their kids graduate from school, I am worried about their life with TIV after kid’s graduation. Most ALS active patients want to pursue their goal to speak out their thoughts in media, but not all the ALS patients are like that. (JP1)
	Requiring the patients’ consent that they can never stop TIV regardless their wish before starting TIV	Before starting TIV, I always ask the patient to confirm that they can never stop TIV, even if they really want to stop it. (JP2)
	Doctor’s weighing on a particular option based on professional perspective at decision-making	Even though it’s not the standard, approach here, I usually do add some color to the discussion of my own opinions with the caveat that, like, you know, obviously, it’s personal. But I think on medical side, we have responsibility to do that because we’re the ones who have medical training. We understand the natural history. We understand the prognosis better. We’ve seen this before. We have way more information. I think it’s unfair not to offer a bit of guidance on, "I think this might be the better option." I think there’s a balance between being the Irish approach, which is, "We made a decision," versus the US approach, which is, "You could pick x, y, or z. They are all equivalent."(US1)
2-d Doctor’s concerns regarding TIV withdrawal under cultural constraints	Doctor’s paternalistic attitude to ALS patients requiring TIV Doctor’s concerns for patient’s misunderstanding between withdrawing TIV and assisted dying	Particular doctors would provide TIV for the patients, and others would not. In the most part of decision-making process, only doctors have critical role for decision-making. I am suspicious that they might give biased information for the patient. (JP4) But with that caveat, it’s not guaranteed when you remove it that somebody will necessarily die quickly and they need to understand that, really. And I think certainly we wouldn’t want to do it if they stated that the reason was that then they would be able to decide when they died. We’d have to sort of talk about that and understand what it was that they were worried about happening. It definitely probably does create the situation really where it’s easier for them to be in control (UK3)
	Legal concern for agreement with the patient’s wish to withdraw TIV	I think that in Japan today, it’s difficult to stop TIV, it’s illegal. I don’t think that all lawyers will necessarily say that if it’s all ethically OK, then it’s legally OK. (JP7)
	Doctors’ expressivist concerns for showing supportive stance for withdrawal of TIV, euthanasia, or assisted dying	If we show our support for withdrawing TIV in public, we may make enemies. Some doctors think that discontinuing TIV is absolutely unacceptable. We have to be careful. (JP6)

## Data Availability

The participants of this study did not give written consent for their data to be shared publicly, so due to the sensitive nature of the research, supporting data is not available.
